# Antidepressant efficacy of sertraline and imipramine for the treatment of major depression in elderly outpatients

**DOI:** 10.1590/S1516-31802000000400005

**Published:** 2000-07-07

**Authors:** Orestes Vicente Forlenza, Alberto Stoppe, Edson Shiguemi Hirata, Rita Cecília Reis Ferreira

**Keywords:** Depression, Elderly, Antidepressant drugs, Tricyclics, Sertraline, SSRI's, Depressão, Idoso, Antidepressivos, Tricíclicos, Sertralina, ISRS's

## Abstract

**CONTEXT:**

Most double-blind studies of efficacy and tolerability of sertraline as compared to tricyclics in the treatment of late-life major depression have used amitriptyline as a standard, leading to the inevitable conclusion that the former drug is better tolerated than the latter, with both being equally efficacious.

**OBJECTIVE:**

To compare the antidepressant efficacy and tolerability of sertraline (50 mg/day) and imipramine (150 mg/day) in the first 6 weeks of the treatment of major depression in the elderly.

**DESIGN:**

A randomized double-blind parallel study with 6 weeks of follow-up.

**SETTING:**

The psychogeriatric clinic at the Institute of Psychiatry, Hospital das Clínicas, Faculty of Medicine of the University of São Paulo.

**PARTICIPANTS:**

55 severe and moderately depressed non-demented outpatients aged 60 years or more.

**INTERVENTION:**

Patients were assigned to sertraline 50 mg/day or imipramine 150 mg/day.

**MAIN MEASUREMENTS:**

CAMDEX interview. Psychiatric diagnosis followed the guidelines for "Major Depressive Episode" according to DSM-IV criteria. Severity of symptoms was evaluated using the "CGI" and "MADRS" scales. Cognitive state was assessed using the Mini-Mental State Examination. Side effects were assessed using the "Safetee-Up" schedule.

**RESULTS:**

Both groups had a significant decrease in depressive symptoms according to the MADRS scores after 6 weeks of treatment (P = 0.01). No significant differences between groups were detected regarding treatment outcome (t = 0.4; P = 0.7). Although the dropout rate was greater in the imipramine group, the overall tolerability among patients who completed the 6-week trial was similar in both test groups.

**CONCLUSIONS:**

Both sertraline and imipramine exhibited good efficacy and an acceptable side-effect profile for elderly depressed patients after 6 weeks of antidepressant treatment.

## RESUMO

**CONTEXTO:**

A maioria dos estudos duplo-cegos sobre eficácia e tolerabilidade da sertralina em comparação aos antidepressivos tricíclicos no tratamento da depressão maior em idosos, tomaram como base a resposta à amitriptilina e conduziram à inevitável conclusão de que a primeira droga é mais bem tolerada do que a última, sendo ambas igualmente eficazes.

**OBJETIVOS:**

Comparar a eficácia antidepressiva e a tolerabilidade da sertralina (50 mg/dia) e da imipramina (150 mg/dia) em pacientes idosos nas primeiras seis semanas de tratamento ambulatorial.

**DESENHO:**

Estudo randomizado, duplo-cego, paralelo.

**LOCAL:**

Ambulatório de psiquiatria geriátrica (Projeto Terceira Idade) do Instituto de Psiquiatria do Hospital das Clínicas da Faculdade de Medicina da Universidade de São Paulo.

**PARTICIPANTES:**

55 pacientes ambulatoriais com 60 anos de idade ou mais, acometidos por episódio depressivo maior de moderada ou grave intensidade (segundo DSM-IV), não-demenciados.

**INTERVENÇÃO:**

Tratamento com sertraline 50 mg/dia ou imipramine 150 mg/dia.

**VARIÁVEIS ESTUDADAS:**

Anamnese com CAMDEX. O diagnóstico psiquiátrico seguiu as recomendações para "episódios depressivos maiores" de acordo com o critério do DSM-IV. Os simtomas de gravidade foram avaliados com as escalas "CGI" and "MADRS". O estado cognitivo foi avaliado pelo "Mini-Mental State Examination". Os efeitos colaterais foram avaliados com a lista "Safetee-Up".

**RESULTADOS:**

Ambosos grupos apresentaram redução significante dos escores para sintomas depressivos segundo a escala de MADRS após 6 semanas de tratamento (P = 0.01); não foram identificadas diferenças estatisticamente significantes entre os grupos no que diz respeito à evolução clínica (t = 0.4; P = 0.7); embora a taxa de abandono tenha sido maior entre os pacientes que receberam imipramina, a tolerabilidade para os pacientes que concluíram seis semanas de tratamento foi comparável nos dois grupos.

**CONCLUSÕES:**

Sertralina e imipramina foram drogas eficazes e satisfatoriamente toleráveis para o tratamento ambulatorial de pacientes deprimidos idosos.

## INTRODUCTION

Most double-blind studies of efficacy and tolerability of sertraline as compared to tricyclics in the treatment of late-life major depression have used amitriptyline as a standard,^1,2^ leading to the inevitable conclusion that the former drug is better tolerated than the latter, with both being equally efficacious. More recently, Finkel et al.^3^ examined such issues in a cohort of 76 outpatients aged 70 or more, in a double-blind randomized study of flexible-dose sertraline (50 to 150 mg/day) or nortriptyline (25 to 100 mg/day), with results that also favored the selective serotonin re-uptake inhibitor (SSRI) in such an older cohort.

Tricyclic antidepressants may be viewed as hazardous drugs for the treatment of late-life depression, especially due to the risk of anticholinergic side-effects, falls related to postural hypotension,^4^ cardiac toxicity,^5^ and cognitive impairment.^6,7^ There is no doubt that the new generation drugs, including the SSRI's and related classes, represent the first therapeutic choice in most cases of mild and moderate depression, due to being safer, better tolerated, and easier to prescribe. However, increasing evidence demonstrates that the latter drugs may also induce severe side effects, such as parkinsonism and serotonin syndrome. In addition, they may be less efficacious in the treatment of psychotic depression, as compared to standard drugs and ECT. For elderly patients with severe depression, or for those who are hospitalized, secondary amine tricyclic antidepressants, such as nortriptyline, are perceived as highly effective treatment.^8^ As these are well tolerated drugs among the tricyclics, they continue to be relied upon and are among the most widely prescribed of such medications.^9-11^

A recent meta-analysis study of efficacy, safety and tolerability of four antidepressant classes concluded there were no differences between outcomes in the management of late-life major depression,^12^ suggesting that there is little advantage for each antidepressant class over the others, namely SSRI's, tricyclics, reversible inhibitors of monoamine oxidase-A and atypical antidepressants. Nevertheless, such finding can only be generalized for samples of selected patients. In the presence of concurrent medical illnesses, or physical and cognitive conditions, accurate clinical judgement must be relied upon for the correct choice of antidepressant, as the use of certain drugs is limited by the risk of toxicity, pharmacokinetic interactions or intolerable side-effects.

Although more risky and laborious to prescribe, the clinician must be aware of the putative benefits of tricyclic antidepressants in the treatment of depressive disorders in the elderly. Besides being effective, tricyclics are cheaper and more widely available than the new class drugs, which are not always accessible in certain settings, especially within primary care facilities or among socially deprived patients. The current study presents efficacy and tolerability data on the use of sertraline and imipramine for treatment of major depression in elderly patients, and we suggest that both drugs can be successfully used to provide adequate treatment for such condition, in accordance with careful clinical judgement and management.

## METHODS

### Ethics

The procedures that follow were in accordance with the ethical standards of the committee responsible for human experimentation and with the Helsinki declaration of 1975, as revised in 1983.

### Design

This was a randomized double-blind parallel study with 6 weeks of follow-up. We present here the preliminary data from the first 6 weeks of treatment. Cases of psychotic depression, as well as suicidal patients, were not included in the study.

### Subjects

Severe and moderately depressed patients aged 60 years or more were clinically evaluated and laboratory tested for concurrent organic diseases. Cerebral disease was ruled out by computer tomography scans. Patients with life-threatening medical conditions, or at risk of severe clinical complications, due to anti-cholinergic effect (such as narrow-angle glaucoma) or to cardiac events were excluded from the trial. Additional exclusion criteria were alcohol or drug-related problems, and previous treatment for depression within the past 2 months.

### Intervention

*Study.* The patients were treated with sertraline 50 mg/day or imipramine 150 mg/day. Patients who terminated prematurely and those who completed the study were all followed in a naturalistic way. Patients who did not respond to sertraline 50 mg/day or imipramine 150 mg/day after 6 weeks were eligible to have an increased dose or, if necessary in the absence of appropriate response, a crossover change of medication.

*Sampling.* Subjects were selected from consecutive referrals to the outpatient service of the Unit for Mental Health in the Elderly at Hospital das Clínicas, São Paulo, Brazil. They were all informed about the details of the study and enrolled only after giving written consent. This project was approved by the Ethics Committee of Hospital das Clínicas. The randomization process was centralized at Pfizer and the code was broken only after completion of the study.

*Allocation concealment and double-blind methods.* Every patient enrolled in the trial was, after each assessment, given a sealed envelope labeled with his or her trial number, containing the medication (b.i.d.) for each day of the period of treatment that followed. Dose titration was necessary for the imipramine group, and double-blindness was preserved by maintaining a fixed daily number of tablets, and varying the quantity of active drug or placebo within each dose. All subjects received the same number of tablets (same shape and color) throughout the trial. Imipramine treatment was started with a daily dose of 25 mg for two days. Increments of 25 mg occurred on days 3, 5, 7, 9, and 11, when the final dose of 150 mg per day was attained. Patients randomized to treatment with sertraline received one tablet of 50 mg of active drug and identical placebo tablets daily from the baseline onwards.

### Main measurements

All subjects in the test groups were assessed using the "CAMDEX" interview.^13^ Psychiatric diagnosis followed the guidelines for "Major Depressive Episode" according to DSM-IV criteria.^14^ Severity of symptoms was evaluated using the "CGI" and "MADRS" scales.^15,16^ In order to include only moderate and severe cases of depression, the cutoff points for these instruments were respectively 4 and 20 (inclusive). Cognitive state was assessed using the "Mini-Mental State Examination",^17^ and cognitive-impaired patients (i.e. MMSE < 24, or < 17 for illiterates) were excluded from the sample. Side effects were assessed using the "Safetee-Up" schedule.^18^ An ECG was performed prior to the administration of treatment, and follow-up exams were requested at the 6th week, in order to investigate minor cardiac changes related to the drugs in use.

Response to treatment was defined as a 50% decrease in the "MADRS" score, and the overall decline was used to compare the efficacy between the two treatment groups.

### Statistical methods

The data were analyzed using the Statistical Package for Social Sciences (SPSS/PC 6.0 for Windows). Likelihood ratio analysis of contingency tables using the Pearson method was used in the investigation of categorical data, with the statistical result being distributed in the chi-squared form (χ2). The Fisher Ex-act Test (FET) was used in the analysis of 2 × 2 tables when 2 or more of the cells had expected values of 5 or less. The odds ratio was calculated to compare the likelihood of subjects reporting adverse events in the two treatment groups. Student's t-test (t) was applied to compare the scores of outcome measurements (the degrees of freedom for the t-tests equal the total number of subjects minus 2, unless stated otherwise).

## RESULTS

### Baseline comparisons

Fifty-five patients were randomly assigned to treatment with sertraline or imipramine after informed consent. Demographic and clinical data of patients who entered the study are presented in Tables 1 and 2. Twenty patients were withdrawn during follow-up.

**Table 1A:** Demographic variables of the patients who accepted participating in the study as a whole and per treatment group

Variables		N = 55	imipramine	sertraline
Age, years.		68.5 (5.9)	69.0 (4.9)	68.0 (6.8)
Gender, %.	Female	38 (69.1)	17	21
	Male	17 (30.9)	10	7
Race, %.	Caucasian	46 (83.6)	22	24
	Negroid	7 (12.7)	4	3
	Asiatic	2 (3.6)	1	1
Marital status, %.	Single	7 (12.7)	5	2
	Married	28 (50.9)	14	14
	Unmarried	4 (7.3)	0	4
	Widowed	16 (29.1)	8	8
Education level, %.	Illiterates	6 (10.9)	4	2
	Primary	22 (40.0)	9	13
	Secondary	11 (20.0)	6	5
	Higher/further	12 (21.8)	6	6

**Table 1B:** Clinical features of the depressive illnesses of the patients in the cohort (n = 55)

First episode	28	(50.9%)	
Late-onset (≥ 60 years)	24	(43.6%)	
Duration (current episode)	12	(21.8%)	< 3 months
	22	(40.0%)	3 months-1 year
	18	(32.7%)	> 1 year
Previous treatment	20	(36.4%)	antidepressants
	3	(05.5%)	ECT
	5	(09.1%)	psychotherapy
Admitted to hospital	3	(05.5%)	
Family history of depression	22	(40.0%)	

**Table 2A:** Severity of the depressive illness at first assessment (t_0_), per group, according to the respective instruments

Instrument	mean (SD) n = 55		Imipramine n = 27		Sertraline n = 28	
CGI	4.4	(0.7)	4.4	(0.7)	4.4	(0.7)
MADRS	29.0	(5.0)	28.1	(5.0)	29.8	(5.0)
MMSE	26.8	(3.6)	26.4	(3.5)	27.3	(3.8)

CGI = Clinical Global Impression; MADRS = Montgomery-Asberg Rating Scale; MMSE = Mini-mental State Examination.

**Table 2B:** Reasons that accounted for early interruption of treatment in each group at six weeks

Reasons	Imipramine (n=27)	Sertraline (n=28)
Side-effects	8 (29.6%)	5 (17.9%)
Medical reasons (not related to drug in use)	1 (3.7%)	1 (3.6%)
Unknown (non-compliant patients)	3 (11.1%)	2 (7.1%)
**Total**	**12 (44.4%)**	**8 (28.6%)**

### Main outcomes

**Figure 1:**
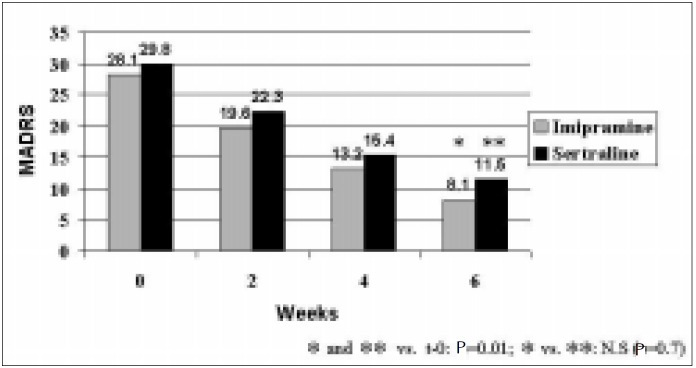
Estimates of treatment outcome according to MADRS (Montgomery-Asberg Rating Scale).

**Figure 2:**
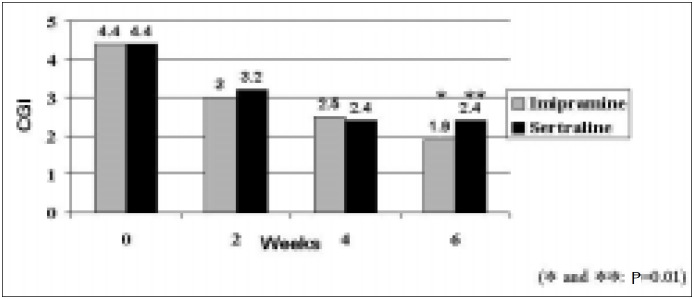
Estimates of treatment outcome according to CGI (Clinical Global Impression).

Treatment outcomes for the 35 patients who completed the trial at week 6 are presented in Figures 1 and 2. Among compliant patients in both groups, there was a significant decrease in MADRS scores after 6 weeks of treatment (P = 0.01). Prior to treatment the imipramine and sertraline groups scored 28.2 (SD 5.0) and 29.8 (SD 5.0) respectively (t = -1.24; P = 0.22). After 6 weeks, treatment scores were 8.1 (SD 8.0) and 11.5 (SD 8.9) respectively (t = -1.18; P = 0.25). There was no significant difference between groups in the decline of depressive symptoms (t = 0.4; P = 0.7). A decrease of at least 50% on MADRS scores was observed in 86.7% and 65.0% of patients on imipramine and sertraline respectively (FET; P = 0.244).

Similar information was obtained by means of an intention to treat analysis, which consisted of the inclusion of the non-compliant patients of each group in the overall efficacy measurements, bringing the last documented score forward. Here again, there were no significant differences between patients receiving imipramine and sertraline, regarding the percentage of patients who experienced a reduction of 50% or more on MADRS scores (60.7 and 55.6% respectively, P = 0.698), or regarding the final MADRS scores of 12.71 (SD 4.6) for the imipramine group and 14.44 (SD 4.9) for the sertraline group (P = 0.598).

**Figure 3:**
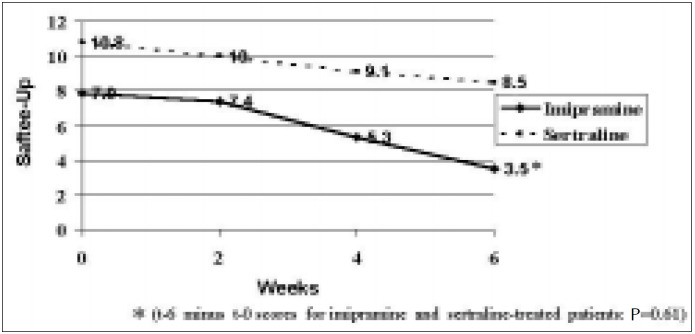
Side-effect scores for each treatment group at first assessment and after 2,4 and 6 weeks, according to the Safetee-Up questionnaire.

**Figure 4:**
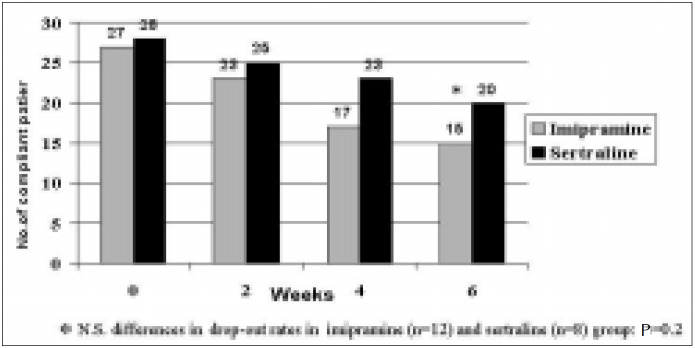
Discontinuation of treatment after 2, 4 and 6 weeks, per group.

The initial Safetee-Up score was 7.96 (SD 4.1) for those on imipramine and 10.79 (SD 8.6) for those on sertraline. After 6 weeks of treatment the scores declined respectively to 4.40 (SD 4.7) and 3.10 (SD 9.0), without significant difference between the two groups (t = 0.51; P = 0.61) (Figure 3). The number of dropouts after the first 6 weeks of treatment was respectively 12 (44.4%) in the imipramine and 8 (28.6%) in the sertraline group (χ2 = 1.5; P = 0.2) (Figure 4), and the respective reasons are listed in table 2B.

Values are showed in mean and standard deviation.

## DISCUSSION

Outcome and tolerability data for 35 compliant patients out of the initial sample of 55 are presented here. Both treatments were efficacious, and there were no statistically significant differences between the two antidepressant classes with respect to efficacy, as measured by a 50% decrease in the MADRS scores, which is in agreement with the literature (57.4 to 76.7% for tricyclics and 45.5 to 69.8% for SSRI's).^12^

In spite of this fact, the dropout rates in this study were particularly high. In general, dropout rates in pharmaceutical trials are high (11 to 27%), independent of the drug class utilized, or even within placebo groups (25.6%).^12^ The high dropout rates can be attributed to many reasons, such as intolerable adverse events, lack of efficacy, medical complications not related to the antidepressant treatment, or unknown causes. The circumstances under which non-compliance occurred were documented, the majority of which were related to side effects. Considering the cases where discontinuation of treatment was clearly a consequence of drug-related side-effects, such percentages are not dissimilar to those found by other authors who compared imipramine and sertraline for the treatment of chronic depression.^19^ The distribution of the other non-completers was similar in the two groups. Social and cultural circumstances may justify some of the cases of discontinuation of treatment for unknown reasons. Among the dropout cases with whom no further contact has been possible, there may be patients who have returned to their homes in distant parts of the country after an initial satisfactory improvement, as well as patients who rejected treatment after experiencing adverse reactions, in spite of having agreed with the consent terms.

Taking into account the possibility of absenteeism at random in both groups, then the response rates would represent an estimate of the biological efficacy for each drug, and the tolerability analysis could be employed to evaluate the observed reactions to the treatment actually received.

However, the exclusion of non-compliers from this preliminary analysis possibly constitutes a source of selection bias. Intention-to-treat analysis was done in order to evaluate this potentially hazardous drawback, and no conflicting information was produced when compared to the analysis of the compliant sample. It must be said, though, that such a cohort may not be a random sample of the original patient groups, in which case confusion between compliance and outcome is present. Furthermore, the selection pattern for compliance may be different between the treatment groups being studied, resulting in interaction between compliance and treatment. The latter circumstance is particularly important in this case. The expected different side-effect profiles for the two drugs being studied are liable to result in a selective removal of patients at higher risk of having more severe adverse events related to one of them. In our test group, the number of patients who discontinued treatment due to drug intolerance was higher in the imipramine group (29.6%) then in the sertraline group (17.9%), although not within significance levels. Such numbers are in line with the dropout rates of the majority of studies available in the literature, i.e. 23.2% for tricyclics and 18.5% for SSRI's.^12^

It is interesting to note that both groups had a significant decline in the side-effect scores as the treatment progressed. This finding suggests that the intensity of such complaints may have been overestimated by the patients at the beginning of the treatment, due to the presence of depressive symptoms. A more detailed investigation of the nature of these complaints is in course and will be presented in the definitive analysis.

Another issue that must be addressed is the fact that the test group included in this trial is composed of healthy non-demented patients. Such subjects might be more likely to tolerate certain side effects better (e.g. anti-cholinergic effects, sedation, hypotension). Moreover, the risk of adverse reactions affecting cognition or cardiovascular function in such cases is definitely smaller than among the demented or the frail elderly, as well as the occurrence of drug interactions between antidepressants and the medications for the treatment of medically ill patients. These situations often contra-indicate the use of tricyclics, and favor the choice of an SSRI in clinical practice.

Most studies of SSRI efficacy and tolerability for the treatment of major depression in the elderly have been based on the comparison with amitriptyline.^1,2^ We understand that the latter drug has, among the TCA group, the most intense anti-cholinergic and sedative profiles, which usually result in undesirable side-effects and increased risk of medical complications. We have chosen imipramine as the representative of the TCA group because it is more likely to be tolerated by the elderly than the former drug, and also because it has been regarded as the gold standard for drug efficacy in other depression trials.

Elderly depressed patients prescribed tricyclics have been shown to have higher overall adverse event rates and higher dropout rates, but higher response rates when compared to SSRI's.^12^ This may indicate that elderly depressed patients may not tolerate the former drugs very well, but when they do tolerate these medications, patients may respond better.

## CONCLUSIONS

For elderly depressed patients who completed a 6-week treatment trial, both sertraline and imipramine exhibited good efficacy and few side effects. There was no difference between groups in the response rate or the severity of side effects due to drug treatment. Many side effects commonly reported among these elderly patients may be due to the presence of depression rather than to specific side effects of the drugs.
